# Clinical Significance of Precipitous Labor

**DOI:** 10.14740/jocmr2058w

**Published:** 2014-12-29

**Authors:** Shunji Suzuki

**Affiliations:** Department of Obstetrics and Gynecology, Japanese Red Cross Katsushika Maternity Hospital, Tokyo, Japan. Email: czg83542@mopera.ne.jp

**Keywords:** Precipitous labor, Hypertensive disorders, Maternal outcome

## Abstract

**Background:**

Precipitous labor is defined as expulsion of the fetus within less than 3 hours of commencement of regular contractions. We retrospectively examined our cases of precipitous labor to identify the clinical significance and perinatal outcomes following precipitous labor in singleton vertex deliveries.

**Methods:**

A retrospective population-based study was conducted comparing women with singleton precipitous labor and those with labor of normal duration. We examined the clinical characteristics and outcomes by comparing patients with precipitous labor and those with labor of normal duration in 0 and two-parous singleton pregnant women.

**Results:**

Using a multivariate analysis, precipitous labor in nulliparous women was independently associated with teenagers (adjusted OR: 1.71, 95% CI: 0.99 - 2.95, P = 0.049), preterm delivery (adjusted OR: 1.77, 95% CI: 1.16 - 2.70, P < 0.01) and hypertensive disorders (adjusted OR: 1.77, 95% CI: 1.19 - 2.65, P < 0.01), while in two-parous women, it was independently associated with hypertensive disorders (adjusted OR: 2.64, 95% CI: 1.33 - 5.24, P < 0.01). No significant differences were noted between the two groups regarding maternal or neonatal complications on both nulliparous and two-parous women.

**Conclusion:**

Although precipitous labor was associated with hypertensive disorders in singleton vertex deliveries, it was not associated with maternal or neonatal outcomes.

## Introduction

Not only can labor be too slow, but it also can be abnormally rapid [1-4]. Precipitous labor is extremely rapid labor and delivery. It is defined as expulsion of the fetus within less than 3 h of commencement of regular contractions [1]. It has been supposed to result from an abnormally low resistance of the soft pass of birth canal, from abnormally strong uterine and abdominal contractions, or rarely from the absence of painful sensations [1]. The prevailing opinion has been that too rapid a labor can result in maternal injury and place the fetus at risk for traumatic or asphyxia insults [1]. For example, the uterus that contracts with unusual vigor before labor may be likely to be hypotonic after delivery, with hemorrhage from the placental implantation as the consequence. Postpartum hemorrhage associated with uterine atony following short labor in multiparous women seems to be experienced often in the clinical setting. In addition, precipitous labor has been observed to be associated with the higher rate of placental abruption [2, 3]. However, limited information exists on maternal and perinatal outcome after precipitous labor, especially in nulliparous women [2, 3]. For example, in an earlier study with 99 precipitous labors at term by Mahon et al [2], precipitous labor occurred mostly in multiparous women. In their study, there were only nine nulliparous women (9.1% of all precipitous delivery) with precipitous delivery.

In this study, therefore, we retrospectively examined our cases of precipitous labor to identify the clinical significance and perinatal outcome following precipitous labor.

## Patients and Methods

A retrospective population-based study was conducted comparing women with singleton precipitous labor and those with labor of normal duration. Labor of normal duration is defined as expulsion of the fetus with 3 - 30 h after commencement of regular contractions in nulliparous women and 3 - 15 h after commencement of regular contractions in parous women, while prolonged labor is defined as expulsion of the fetus more than 30 h after commencement of regular contractions in nulliparous women and more than 15 h after commencement of regular contractions in parous women [5]. Deliveries in this study occurred between the years 2009 and 2013 in the Japanese Red Cross Katsushika Maternity Hospital, one of main Perinatal Centers in Tokyo, Japan. In our hospital, oxytocin has not been used in the routine for prevention of postpartum hemorrhage in cases of precipitous labor. Data were collected from the patients’ charts that consist of information collected directly after delivery by the midwives who examine the information routinely before entering it into the database. The following clinical characteristics and outcomes were analyzed: parity; maternal age; gestational age; birth weight; maternal complications such as hypertensive diseases, glucose intolerance; placental abruption; oxytocin use; delivery modes; neonatal Apgar score at 1 and 5 min; umbilical artery pH; postpartum hemorrhage requiring hemotransfusion; perineal tears (severe perineal laceration: perineal laceration either third- or fourth-degree laceration); and cervical tears.

In this study, we examined these clinical characteristics and outcomes by comparing patients with precipitous labor and those with labor of normal duration in 0 and two-parous women.

Statistical significance was ascertained using the *X*^2^ test or Fisher’s exact test for differences in qualitative variables and the Student’s *t*-test for differences in continuous variables. A multivariate logistic regression model, with backward elimination, was constructed in order to find independent factors associated with precipitous labor. Odds ratios (ORs) and their 95% confidence intervals (CIs) were computed. P < 0.05 was considered statistically significant.

## Results

During the study period, there were 11,239 singleton deliveries in our hospital as shown in Table 1. Of these, 1,606 (14.3%) were precipitous deliveries. The incidence of precipitous labor in the parous women (21.5%) was significantly higher than that in the nulliparous women (6.9%, OR: 3.66, 95% CI: 3.24 - 4.13, P < 0.01).

**Table 1:** Incidences of Precipitous and Prolonged Singleton Labors

Parity	Total	Vaginal labor			Cesarean delivery
		Precipitate labor	Normal	Prolonged labor	
0	5,555	386 (6.9)	3,776 (68.0)	29 (0.5)	1,364 (24.6)
1	3,880	817 (21.1)	1,844 (47.5)	48 (1.2)	1,171 (30.2)
2	1,308	320 (24.5)	590 (45.1)	8 (0.6)	390 (29.8)
≥ 3	496	83 (16.7)	277 (55.8)	3 (0.6)	133 (26.8)
Total	11,239	1,606 (14.3)	6,487 (57.7)	88 (0.8)	3,058 (27.2)

Table 2 shows clinical characteristics and outcomes of the two groups of nulliparous women with precipitous and normal labor. The women with precipitate deliveries were more likely to be younger, at lower gestational age of delivery, without oxytocin use, lower birth weight of infant, and hypertensive disorders as compared to those without precipitate deliveries. Using a multivariate analysis, precipitous labor was independently associated with teenagers (adjusted OR: 1.71, 95% CI: 0.99 - 2.95, P = 0.049), preterm delivery (adjusted OR: 1.77, 95% CI: 1.16 - 2.70, P < 0.01) and hypertensive disorders (adjusted OR: 1.77, 95% CI: 1.19 - 2.65, P < 0.01). No significant differences were noted between the two groups regarding maternal or neonatal complications as shown in Table 2.

**Table 2:** Clinical Characteristics and Outcomes in Nulliparous Women With and Without Precipitate Labor

	Precipitate labor	Normal group	P-value	Crude OR	95% CI
Total	386	3776			
Maternal age					
< 20 years	21 (5.4)	115 (3.0)	0.01	1.85	1.14 - 3.00
20 - 34 years	263 (68.1)	2,670 (70.7)	-	Reference	-
≥ 35 years	101 (26.2)	991 (25.2)	0.78	1.03	0.81 - 1.32
Gestational age at delivery					
< 37 weeks	53 (13.7)	159 (4.2)	< 0.01	3.47	2.49 - 4.84
37 - 40 weeks	293 (75.9)	3,050 (80.8)	-	Reference	-
41 - 42 weeks	40 (10.3)	563 (14.9)	0.08	0.74	0.53 - 1.04
Hypertensive disorders	36 (9.3)	174 (4.6)	< 0.01	2.13	1.46 - 3.10
Glucose intolerance	10 (2.6)	87 (2.3)	0.72	1.13	0.58 - 2.19
Oxtocin use	106 (27.5)	1,353 (35.8)	< 0.01	0.68	0.54 - 0.86
Placental abruption	4 (1.0)	58 (1.5)	0.44	0.67	0.24 - 1.86
Vacuum/forceps delivery	56 (14.1)	411 (12.2)	0.05	1.35	1.00 - 1.82
Neonatal birth weight					
< 2,500 g	110 (28.5)	349 (9.2)	0.01	3.64	2.84 - 4.67
2,500 - 3,499 g	293 (75.9)	3,039 (80.5)	-	Reference	-
≥ 3,500 g	40 (10.3)	388 (10.3)	< 0.01	0.39	0.22 - 0.68
Apgar score at 1 min < 4	0 (0)	14 (0.4)	0.23	-	-
Apgar score at 5 min < 7	1 (0.3)	7 (0.2)	0.75	1.40	0.17 - 11.40
Umbilical artery pH < 7	0 (0)	10 (0.3)	0.31	-	-
Total blood loss ≥ 1,000 mL	28 (7.3)	319 (8.4)	0.42	0.85	0.57 - 1.27
Hemotransfusion	4 (1.0)	18 (0.5)	0.15	2.19	0.74 - 6.49
Cervical laceration	12 (3.1)	66 (1.3)	0.06	1.8	0.97 - 3.37
Severe perineal laceration	14 (3.6)	141 (3.7)	0.91	0.97	0.55 - 1.70

Values are expressed as number (percentage). OR: odds ratio; CI: confidence interval. Severe perineal laceration, perineal laceration either third- or fourth-degree laceration.

Table 3 shows clinical characteristics and outcomes of the two groups of two-parous women with precipitous and normal labor. The women with precipitate deliveries were more likely to have oxytocin use and hypertensive disorders as compared to those without precipitate deliveries. Using a multivariate analysis, precipitous labor was independently associated with hypertensive disorders (adjusted OR: 2.64, 95% CI: 1.33 - 5.24, P < 0.01). No significant differences were noted between the two groups regarding maternal or neonatal complications as shown in Table 3.

**Table 3:** Clinical Characteristics and Outcomes in Two-Parous Women With and Without Precipitate Labor

	Precipitate labor	Normal group	P-value	Crude OR	95% CI
Total	320	590			
Maternal age					
< 20 years	0 (0)	1 (0.2)	0.47	-	-
20 - 34 years	169 (52.8)	326 (55.3)	-	Reference	-
≥ 35 years	151 (47.2)	263 (44.6)	0.46	1.11	0.84 - 1.46
Gestational age at delivery					
< 37 weeks	21 (6.6)	30 (5.1)	0.28	1.37	0.77 - 2.44
37 - 40 weeks	261 (81.6)	511 (86.6)	-	Reference	-
41 - 42 weeks	38 (11.9)	49 (8.3)	0.07	1.52	0.97 - 2.38
Hypertensive disorders	30 (9.4)	15 (2.7)	< 0.01	3.71	1.99 - 6.92
Glucose intolerance	10 (3.1)	15 (2.5)	0.61	1.24	0.55 - 2.79
Oxtocin use	35 (10.9)	35 (5.9)	< 0.01	1.95	1.19 - 3.18
Placental abruption	4 (1.3)	4 (0.7)	0.38	1.85	0.46 - 1.47
Vacuum/forceps delivery	9 (2.8)	16 (2.7)	0.97	1.01	0.44 - 2.32
Neonatal birth weight					
< 2,500 g	28 (8.8)	42 (7.1)	0.35	1.27	0.77 - 2.10
2,500 - 3,499 g	247 (77.2)	470 (79.7)	-	Reference	-
≥ 3,500 g	45 (14.1)	48 (13.2)	0.65	1.1	0.74 - 1.63
Apgar score at 1 min < 4	0 (0)	1 (0.2)	0.46	-	-
Apgar score at 5 min < 7	0 (0)	0 (0)	-	-	-
Umbilical artery pH < 7	0 (0)	0 (0)	-	-	-
Total blood loss ≥ 1,000 mL	17 (5.3)	32 (5.4)	0.94	0.98	0.53 - 1.79
Hemotransfusion	0 (0)	0 (0)	-	-	-
Cervical laceration	3 (0.9)	8 (1.4)	0.58	0.69	0.18 - 2.61
Severe perineal laceration	0 (0)	3 (0.5)	0.2	-	-

Values are expressed as number (percentage). OR: odds ratio; CI: confidence interval. Severe perineal laceration, perineal laceration either third- or fourth-degree laceration.

## Discussion

The incidence of precipitous labor in this study was about 14% of all singleton deliveries, which seemed to be higher than those reported in the United States and other countries: 0.1-3% [1-3]. In Japan, the onset of labor is usually defined by the patient’s report of the commencement of regular contractions of 10-min intervals. The high incidence in this study may be attributed to the different definitions as well as the diagnosis of regular contractions by monitoring and not patient’s report in other countries. In this study, in addition, the incidence of precipitous labor in the nulliparous women was higher than that in parous women, as it is known that generally parous patients have shorter deliveries. This result was supported by some previous studies [1-3].

In some previous studies [2, 3], one of main perinatal complications associated with precipitous labor has been reported to be placental abruption, because placental abruption can cause tachysystole, and thus shorter deliveries. On the contrary, we did find the high incidence of placental abruption in the women with precipitous labor. The reason leading to the different outcomes is not clear. However, our medical policy performing cesarean section immediately after the diagnosis of placental abruption might have attributed to reduce the rate of vaginal delivery in cases of placental abruption [6]. In this study, although the incidence of placental abruption was not associated with precipitous labor, hypertensive disorders seemed to attribute to the increased odds of precipitous labor. Recently, the deficiency of maternal immune response to the fetus that may result in preeclampsia or in pregnancy loss has been discussed as a rejection to the fetus [7, 8]. Therefore, in hypertensive disorders such as preeclampsia, the pathophysiology of rejection to the fetus may be able to cause effective uterine contractions for early expulsion of the fetus. Otherwise, the hypertensive disorders might be a preliminary stage of the placental abruption.

In this study, teenagers and preterm delivery were associated with precipitous labor in nulliparous women. The soft residence of birth canal in young women may cause rapid delivery and prevent the extensive lacerations of the cervix, vagina, vulva or perineum. The smallness of infants in preterm delivery may also prevent these lacerations. In addition, the Japanese women seemed to wish to take cesarean delivery in cases of preterm labor that can take a long time as expected, and these trends might have contributed to the current results.

In an earlier study by Sheiner et al [3], precipitous labor was associated with some maternal complications including perineal lacerations, postpartum hemorrhage, retained placenta, hemotransfusion and prolonged hospitalization. These maternal complications have been previously reported as possible consequences of precipitous labor due to abnormally strong uterine contractions combined with non-dilated cervix and highly resistant birth canal [1, 3]. On the contrary, in this study precipitous labor seemed to be not associated with adverse maternal outcomes. The high incidence of teenagers, small infants and preterm delivery in the precipitous labor of this study may contribute to the unchanged incidence of maternal complications due to maternal soft birth canal and/or small fetus passing through. Otherwise, the current outcomes may be attributed to the different definitions of the commencement of regular contractions between some studies.

In this study, at last, precipitous labor was not associated with maternal or neonatal outcomes. The current result supports the previous observations [1-3].

### Conclusion

Based on the current results, although precipitous labor was associated with hypertensive disorders in singleton vertex deliveries, it was not associated with maternal or neonatal outcomes.
